# The Genetic Landscape of Parkinsonism-Related Dystonias and Atypical Parkinsonism-Related Syndromes

**DOI:** 10.3390/ijms22158100

**Published:** 2021-07-28

**Authors:** Monica Diez-Fairen, Pilar Alvarez Jerez, Joos Berghausen, Sara Bandres-Ciga

**Affiliations:** Laboratory of Neurogenetics, National Institute on Aging, National Institutes of Health, Bethesda, MD 20892, USA; monicadifa@gmail.com (M.D.-F.); pilar.alvarezjerez@nih.gov (P.A.J.); bjoos@yahoo.com (J.B.)

**Keywords:** parkinsonism, dystonia, progressive supranuclear palsy, multiple system atrophy, corticobasal degeneration

## Abstract

In recent decades, genetic research has nominated promising pathways and biological insights contributing to the etiological landscape of parkinsonism-related dystonias and atypical parkinsonism-related syndromes. Several disease-causing mutations and genetic risk factors have been unraveled, providing a deeper molecular understanding of the complex genetic architecture underlying these conditions. These disorders are difficult to accurately diagnose and categorize, thus making genetics research challenging. On one hand, dystonia is an umbrella term linked to clinically heterogeneous forms of disease including dopa-responsive dystonia, myoclonus-dystonia, rapid-onset dystonia-parkinsonism and dystonia-parkinsonism, often viewed as a precursor to Parkinson’s disease. On the other hand, atypical parkinsonism disorders, such as progressive supranuclear palsy, multiple system atrophy and corticobasal degeneration, are rare in nature and represent a wide range of diverse and overlapping phenotypic variabilities, with genetic research limited by sample size availability. The current review summarizes the plethora of available genetic information for these diseases, outlining limits and future directions.

## 1. Introduction

**Figure 1:**
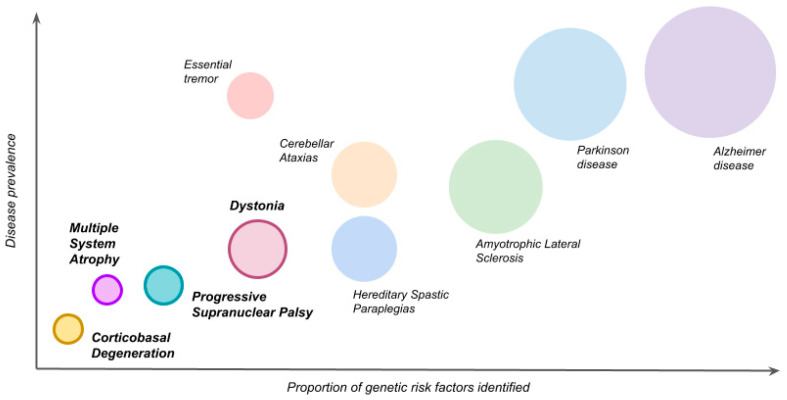
The genetic landscape of movement and neurodegenerative disorders. The diameters of each circumference approximate their relative frequency (greater diameter, greater prevalence).

In the last 20 years, tremendous advances have been made in an attempt to unravel the genetics of rare neurological disorders such as parkinsonism-related dystonias and atypical parkinsonism-related syndromes. The genome-wide association studies (GWAS) era has led to increased collaboration across independent research groups, serving as discovery engines and allowing the identification of new and overlapping genetic loci influencing these conditions. The genetic contribution to these diseases is broad (Figure 1), spanning the etiological risk spectrum from monogenic to more complicated sporadic forms. Clinical signs and neuropathological findings in inherited neurodegenerative disorders are frequently indistinguishable from those of sporadic cases, suggesting that converging genomic pathways and pathophysiologic mechanisms underlie both hereditary and sporadic forms of disease.

An accurate diagnosis of these heterogeneous and clinically complex conditions is challenging, often representing a wide range of diverse and overlapping symptomatology, phenotypic and neuropathology variabilities, and the absence of disease-specific diagnostic tests. Treatments are just symptomatic, focused on ameliorating the motor disturbances. Despite the efforts conducted thus far, our knowledge about the underlying mechanisms contributing to the pathophysiology of these diseases is still scarce, and a lot of work needs to be carried out to accelerate the development of therapeutic approaches.

This review provides our current scenario on the genetic basis of rare forms of parkinsonism, including parkinsonism-related dystonias and atypical parkinsonism-related syndromes, highlighting key genetic advances achieved in the past, and what the present and future hold. We focus on dopa-responsive dystonia, rapid-onset dystonia-parkinsonism and dystonia-parkinsonism. In the atypical parkinsonism-related syndromes arena, we discuss our current understanding on progressive supranuclear palsy, multiple system atrophy and corticobasal degeneration. We highlight challenges, considerations and possible future directions in our continuous and long journey to uncover the molecular complexity underlying these devastating diseases.

## 2. Parkinsonism-Related Movement Disorders

### Dystonia-Plus Syndromes

Historically, a large group of heterogeneous movement disorders have been gathered under the term dystonia, adding considerable clinical and genetic heterogeneity to the definition of dystonia. Dystonia is one of the most common movement disorders after Parkinson’s disease (PD) and essential tremor [1,2]. It is a hyperkinetic movement disorder characterized by sustained or intermittent muscle contractions causing abnormal repetitive movements and postures, often with no structural brain abnormalities [3]. Its clinical presentation ranges from an isolated clinical feature—isolated or primary dystonia—to multi-systemic disorders where dystonia is only a co-occurring sign together with other neurological deficits, particularly in disorders that manifest parkinsonism [4]. The existence of a disease spectrum for dystonias is clear, going from more severe, often monogenic, early-onset dystonias to adult-onset focal primary torsion dystonias, which might develop as a result of a greater interaction between genetic and environmental factors.

Dystonia is known to coexist with parkinsonian disorders and may indeed be a risk factor for parkinsonism [5,6]. Dystonia can be seen in 30% or more of patients with PD, and it is more prevalent in young-onset PD [7], especially in autosomal recessive genetic parkinsonism, as seen in carriers of the parkin (*PARK2)* and *PINK1* mutations [8,9]. Indeed, dystonia can precede PD clinical symptoms by almost a decade [6,10]. From the genetics perspective, parkinsonism is commonly seen in carriers of mutations in DYT genes, such as *TAF1* (DYT3), *ATP1A3* (DYT12) and *PRKRA* (DYT16), but especially in those involved in the dopamine synthesis pathway, *GCH1* (DYT5a) and *TH* (DYT5b) [11]. Apart from the occurrence of dystonia in classical PD, typically affecting the feet or legs [5], facial or cervical dystonia is quite common in patients with multiple system atrophy [12]. Patients with progressive supranuclear palsy can have brachial dystonia or apraxia of eyelid opening, which is indeed considered a form of dystonia [13].

Despite the fact that the genetic basis of many forms of dystonia has been identified (Table 1), the mechanisms by which brain dysfunction results in dystonia are not fully understood. Clear monogenic inheritance usually occurs in more complex, severe forms of dystonia, whereas potential genetic susceptibility factors play a substantial role in primary focal dystonias [11,14,15]. As almost all known forms of dystonia are inherited in an autosomal dominant manner, unlike in parkinsonism, the mode of transmission is not a useful feature to categorize familial dystonias. In contrast, the genetics of late-onset dystonias are complex and, in most cases, seem to be sporadic. Additionally, copy number variants have also been described in dystonia [16].

Dopa-responsive dystonia (DRD) is a rare form of dystonia classically characterized by an excellent patient response to low doses of levodopa and no evidence of motor fluctuations or levodopa-induced dyskinesias, nor presynaptic nigrostriatal degeneration [17]. Parkinsonism can develop later or can be an early feature in adult-onset cases. The most common cause of DRD is a GTP-cyclohydrolase 1 deficiency which is caused by heterozygous mutations in the *GCH1* gene [18]. The gene encodes the enzyme GTP-cyclohydrolase 1 that catalyzes the first step in the biosynthesis of tetrahydrobiopterin, the essential cofactor for tyrosine hydroxylase, which is the rate-limiting enzyme for dopamine synthesis. A recent study by Mencacci and colleagues described that rare *GCH1* coding variants are associated with a seven-fold increase in the risk of PD [19]. Indeed, in recent PD studies and meta-analyses, *GCH1* appears to be a low-risk susceptibility locus for PD [20,21]. Other studies confirmed that parkinsonism is indeed relatively common in *GCH1* mutation carriers (27%) and that the frequency of parkinsonism is dependent on the age of onset of dystonia, being more frequent in carriers with disease onset after the age of 15 years [22]. *GCH1* mutations can present with reduced penetrance, especially in males, which represents a challenge for counselling the families of mutation carriers and complicates the evaluation of the potential pathogenicity of new *GCH1* variants [23,24]. Other rare forms of DRD are caused by autosomal recessive mutations in the *TH* gene, encoding tyrosine hydroxylase itself [25,26].

Rapid-onset dystonia-parkinsonism (RDP) is a rare condition that has a childhood or early adulthood onset with dystonic spasms, bradykinesia, postural instability and dystonic spasms, among others, followed by little progression. Affected individuals can be asymptomatic for years but rapidly develop persistent dystonia and parkinsonism-like symptoms after a stressful experience. Missense mutations in the gene encoding the Na+/K+-ATPase 3 subunit (*ATP1A3; DYT12*) have been identified in patients with RDP [27]. Mutations seem to decrease the activity of the Na+/K+ pump, which is crucial for maintaining the electrochemical gradient across the cell membrane. A mouse model study showed that altered cerebellum activity was the primary instigator of dystonia by altering the basal ganglia function and could account for the symptoms seen in RDP patients [28].

Mutations in the stress response gene *PRKRA* (DYT16), which encodes the protein kinase interferon-inducible double-stranded RNA-dependent activator, were found to cause an autosomal recessive young-onset form of dystonia-parkinsonism disorder in two Brazilian families [29]. Patients had progressive, generalized early-onset dystonia with axial muscle involvement, oromandibular and laryngeal dystonia and, in some cases, parkinsonian features and did not respond to levodopa therapy. These findings have been confirmed in other populations [30,31,32], confirming the causal contribution of the *PRKRA* gene to dystonia-parkinsonism disorders [33].

Dopamine transporter deficiency syndrome is the first identified parkinsonian disorder caused by genetic alterations of the dopamine transporter. This autosomal recessive disorder manifests in infancy with severe parkinsonism-dystonia associated with an eye movement disorder and pyramidal signs. Loss-of-function mutations—both homozygous and compound heterozygous—were identified in the gene *SLC6A3* encoding the dopamine transporter [34].

Another rare adult-onset condition is X-linked dystonia-parkinsonism (XDP) caused by a founder mutation present in the Philippines that is a ~2.6kb SINE-VNTR-Alu (*SVA*)-type retrotransposon insertion in intron 32 of the *TAF1* gene [35]. It is characterized by striatal neurodegeneration, and it is inherited in an X-linked recessive manner, mostly affecting men. XDP shows considerable variability in age, the site of disease onset, initial symptoms and the rate of progression. The length of a polymorphic (*CCCTCT*)_n_ repeat within the *SVA* retrotransposon insertion was shown to inversely correlate with age at onset (AAO) and *TAF1* expression, and to positively correlate with disease severity and cognitive dysfunction [36,37]. Three additional genetic modifiers of AAO for XDP—rs245013 and rs33003 in *MSH3* and rs62456190 adjacent to the *ANKRD61, EIF2AK1* and *PMS2* genes—have been described [38]. Together with the hexanucleotide repeat, these signals account for nearly two thirds of the AAO variability in XDP.

Other monogenic isolated dystonias may also be accompanied by signs of parkinsonism, exemplifying the complex relationship between dystonia and parkinsonism. For example, patients with DYT1 dystonia often misdiagnosed as PD due to a prominent dystonic tremor [39,40] or patients with myoclonus-dystonia due to mutations in the *SGCE* gene that exhibit parkinsonian features may even have a good response to high-dose levodopa treatment [41].

The relatively frequent occurrence of parkinsonism after long-standing dystonia could suggest common dopaminergic pathophysiological mechanisms, which supports evidence from clinical reports, imaging studies, animal models and genetics, as briefly explained above. As dystonia can be the initial manifestation of parkinsonism syndromes, and, conversely, patients with dystonia can present with isolated parkinsonism, it can be difficult to diagnose patients based solely on clinical observations, highlighting the importance of genetic testing in identifying the causes of these complex movement disorders.

## 3. Atypical Parkinsonism-Related Neurodegenerative Disorders

### 3.1. Progressive Supranuclear Palsy

Progressive supranuclear palsy (PSP) is a rare neurodegenerative disease affecting movement, gait and balance, speech, swallowing, vision, mood and behavior. While PSP is often confused with PD in its early stages, PSP is unique due to its ocular signs, most notably supranuclear gaze palsy [42]. Pathologically, PSP is characterized as a tauopathy with an accumulation of tau proteins in the brain. Tau proteins make up a group of six isoforms, achieved by the alternate splicing of exons 2, 3 and 10 of the *MAPT* gene. Specifically, splicing of exon 10 leads to a three (3R)- or four (4R)-repeat tau protein [43]. In a healthy adult brain, these two isoforms are found in equal amounts [44]. However, neurofibrillary tangles (NFTs) found in the brain, a pathological hallmark of PSP, contain an excess of the 4R isoform, causing an increased 4R/3R tau isoform ratio, which is also a pathological characteristic of PSP [45,46]. A recent study conducted in the Japanese population estimated that the prevalence of PSP was 17.9 per 100,000 individuals [47]. 

While most PSP cases happen sporadically, a small number of cases have been linked to a genetic cause. PSP GWAS analyses, for example, have found multiple risk loci, most notably in the Microtubule Associated Protein Tau (*MAPT*) gene [48,49,50]. Family studies have gone on to propose an autosomal inheritance pattern [51,52]. One study estimated that about 6% of PSP cases had an autosomal dominant pattern of inheritance; however, no risk mutations were found in this study’s cohort, and it thus cannot rule out that the percentage could change based on mutation status [53]. It has also been reported that PSP patients are more likely to have first-degree relatives with parkinsonism compared to controls, leading to the hypothesis of a familial aggregation of parkinsonism [54]. Due to the variable clinical presentation [55], there may be other gene associations yet to be discovered for PSP.

The *MAPT* gene, located in chromosome 17, is responsible for the production of protein tau, a microtubule-assembling protein that is key in the nervous system. Based on a 2019 GWAS, *MAPT* contained the two risk loci with the largest effect sizes on PSP (*p* = 1.5 × 10^−116^ and *p* = 4.2 × 10^−70^, respectively) [55]. A previous review estimated the frequency of PSP cases carrying an *MAPT* mutation to be between 0.6% and 14.3%, with a mean AAO of around 44.8 years old, earlier than the idiopathic AAO [56]. Most of the *MAPT* mutations described are located in exon 10, at or near a splicing site, which leads to an overproduction of the 4R tau isoform in the brain. Additionally, three other mutations have been described in exons 1, 12 and 13. The most common genetic allele of *MAPT* associated with PSP is *MAPT-N279K*, with 10 cases described to date [57,58,59]. The mutation *MAPT-K298_H299insQ* is the second most common, with three familial PSP cases described in the Japanese population, being the first report of an insertion variant in the *MAPT* gene [60]. 

Family history appears in most *MAPT* mutation-caused cases and is varied, although a positive history of parkinsonism or dementia is most common. Additionally, there have also been multiple reports of families with autosomal dominant PSP in which one of the individuals carried an *MAPT* mutation, causing amino acid residue substitutions, i.e., L284R or G303V [51,52]. A controversial body of literature exists regarding the contribution of *MAPT* mutations to disease etiology and potential phenotype–genotype correlations. For example, Ogaki et al. suggested that an AAO before 50 and early falling could be indicative of *MAPT* mutations even without a family history [59]. On the contrary, Fujioka et al. proposed that an absence of early falls may be indicative of an *MAPT* mutation or that, at least, falling may be different at an individual mutation level [61].

*LRRK2,* a gene located in chromosome 12, encodes Leucine Rich Repeat Kinase 2. Mutations in this gene are one of the most common genetic causes of PD, with a higher prevalence in groups of Ashkenazi Jewish descent [62]. Although it was not part of a high-risk locus in the PSP GWAS, *LRRK2* has been linked to PSP cases on multiple instances, with five mutations reported. On the other hand, a recent survival-focused GWAS found that an *LRRK2* SNP, rs2242367, had an association with PSP survival for all cohorts included (*p* = 1.3 × 10^−10^) and thus hypothesized that variation in the *LRRK2* locus is a genetic determinant of survival in PSP [63].

The most common mutation in exon 41 is that resulting in the G2019S substitution, with four PSP cases reported [64,65,66,67]. Apart from this mutation, four additional *LRRK2* mutations have been linked to PSP. Of these, there are two mutations in exon 31, including those causing R1441C and R1441H substitutions [68,69,70], one in exon 30, resulting in A1413T [66], and one in exon 41, resulting in T2310M substitutions [71]. Of note, the R1441C substitution was found in 10 affected members of a large kindred family with a clinical diagnosis of parkinsonism in which one of the members had a potential PSP pathology [68]. Compared to the *MAPT* cases, *LRRK2* carriers have a later AAO, around 75 years. A common theme throughout some of the PSP/parkinsonism cases reported with an *LRRK2* mutation was unresponsiveness to levodopa, differing from a regular PD diagnosis [64,65]. Although finding *LRRK2* mutations in PSP patients is rare and needs to be studied more, there is increasing evidence for a causal relationship between *LRRK2* and PSP.

Other genes have also been linked to PSP or PSP look-alike symptoms. Some of these include DCTN1 [72,73,74,75], PARK2 [76,77], BSN [75], GRN [75], C9ORF72 [78,79,80], TARDBP [81], NPC1 [82,83], PGRN [84] and TBK1 [85]. Out of these listed genes, DCTN1 has the largest number of potentially PSP-associated mutations. However, the relationship between genetic mutations and PSP remains unclear as patients are heterogeneous in nature, with a wide variety of pathologies and clinical presentations.

A recent study carried out a phenotype GWAS comparing Richardson syndrome (RS)—the most classic phenotype of PSP—cases versus non-RS cases in two independent PSP cohorts and found an intronic variant (rs564309) in the *TRIM11* gene acting as a genetic modifier of the PSP phenotype [86]. TRIM11 has a critical role in the clearance of misfolded proteins via the ubiquitin proteasome system (UPS), thus linking UPS to tau pathology, which, eventually, could be a potential target for disease-modifying therapies. 

### 3.2. Multiple System Atrophy

Multiple system atrophy (MSA) is a sporadic, extremely progressive and fatal alpha-synucleinopathy [87,88]. The average AAO is 52 to 59 years, with an average survival rate of 7 to 9 years and a yearly incident rate of 3 to 4 per 100,000 individuals [87]. Clinically, MSA is associated with autonomic failure, parkinsonism and ataxia. Typical pathological hallmarks include glial cytoplasmic inclusions (GCI), consisting of misfolded, fibrillated and aggregated alpha-synuclein in the terminal ends of oligodendrocytes. This hallmark differentiates MSA from other typical alpha-synucleinopathies, including PD and Lewy body dementia, where alpha-synuclein is located in neuron bodies. MSA is a challenging disease to study since it is commonly misdiagnosed and has a low prevalence, with a reported heritability between 2.09% and 6.65% [89].

MSA is classified into two major subtypes: parkinsonian (MSA-P) and cerebral (MSA-C). MSA-P is associated with striatonigral degeneration, whereas MSA-C is associated with olivopontocerebellar degeneration. The MSA subtype prevalence is population-specific, being MSA-P-predominant in Caucasian populations (around 70% of cases) and MSA-C in Asian populations [87,88]. Currently, no causative gene has been linked to MSA, classifying it as a sporadic disease. However, many studies have associated MSA with various genetic factors [90].

Since MSA is considered an alpha-synucleinopathy, considerable focus has been placed on the *SNCA* gene, which encodes for alpha-synuclein. Although the *SNCA* gene has been found to be one of the causal genes for PD, its causality with MSA is still unclear [87]. Later studies investigated whether single-nucleotide polymorphisms (SNPs) on the *SNCA* gene were associated with MSA etiology. A GWAS conducted in 2009 analyzed 413 Caucasian MSA cases and 3974 white control samples, identifying two *SNCA* SNPs to be significantly associated with an increased genetic risk for MSA, one in intron 4 (rs3857059) and one downstream of *SNCA* (rs11931074) [91]. Another GWAS study which enrolled 239 cases and 617 control samples of European ancestry found two additional *SNCA* SNPs (rs3822086 and rs3775444) associated with an increased genetic risk for MSA [92]. However, a recent follow-up study was not able to significantly associate rs3822086 and rs3775444 with an increased genetic risk for MSA in the Chinese population [93]. This suggests that SNPs on *SNCA* might only increase the risk of MSA in the European population.

A recent study of multiple Japanese families revealed a common variant (V393A) in *COQ2* (coenzyme-Q2-polyprenyl transferase) as possibly associated with sporadic MSA [94]. This finding was supported by Zhao et al.’s case–control study on Han Chinese MSA patients and meta-analysis with data from Japan, South Korea and China [95]. However, a later GWAS study was not able to replicate an association with *COQ2* and MSA in a European and Northern American cohort [96]. This suggests that *COQ2* V393A may only increase the risk of developing MSA in East Asian populations.

A more recent study found an association between MSA and the Glucocerebrosidase (*GBA*) gene, the major genetic predisposing risk factor linked to PD and responsible for Gaucher disease. The study sequenced the coding regions and flanking splice sites of *GBA* in 969 MSA patients, of which 574 were Japanese, 223 were European and 172 were of North American ancestry, and 1509 control samples, of which 900 were Japanese, 315 were European and 294 were of North American ancestry. The findings showed a carrier frequency of *GBA* variants of around 1.75% in the combined Japanese, European and North American cohorts. Interestingly, *GBA* variants were also significantly more associated with the MSA-C subtype [97]. An additional study was able to find similar results when multiple *GBA* variants (N370S, T369M and R496H) were found in 4 out of 17 autopsy-confirmed MSA cases, of which 6 were of Ashkenazi Jewish descent, 7 were non-Jewish Caucasian European, 1 was Japanese and 3 were of unknown non-Jewish ethnicity [98]. Among the four MSA cases carrying *GBA* variants, an individual carried N370S in the homozygous state, and the remaining three were all heterozygous carriers of N370S, T369M and R496H. However, another study consisting of 118 neuropathologically confirmed British MSA cases showed no association between MSA and *GBA* mutations [99]. The conflicting results depict a lack of clarity around the association of *GBA* variants with MSA. Some of these studies were limited by their small sample size and clinical heterogeneity; therefore, future research in larger cohorts to dissect the role of *GBA* in MSA etiology is guaranteed.

### 3.3. Corticobasal Degeneration

Corticobasal degeneration (CBD) is an extremely rare and sporadic neurodegenerative disease [100]. The average age of onset is 40 to 70, with an average survival rate of seven years and a yearly incident rate of 0.02 per 100,000 individuals [101]. CBD is characterized by a significant accumulation of microtubule-associated protein tau, making it a tauopathy. These tau inclusions are located in neurons and glia in the cortex, basal ganglia, diencephalon and rostral brainstem [101]. Clinically, CBD causes a variety of motor symptoms, the most common being limb rigidity, bradykinesia, limb dystonia and postural instability. The symptoms are also accompanied by several higher cortical dysfunctions, including cognitive impairment, behavioral changes and limb apraxia [102,103]. With such a wide variety of clinical features, misdiagnosis of CBD is very common, to the point that only 25% to 56% of CBD cases are predicted antemortem. Therefore, most CBD cases are autopsy-confirmed postmortem, making studying genetic risk factors increasingly difficult due to small sample sizes.

A GWAS conducted in 2015 comprising 152 autopsy-confirmed CBD samples and 3311 control samples of European ancestry identified significant associations at *MAPT* (*p* = 1.42 × 10^−12^), *lnc-KIF13B-1*, a long non-coding RNA (rs643472; *p* = 3.41 × 10^−8^), and *SOS1* (rs963731; *p* = 1.76 × 10^−7^)[104]. As this GWAS had a limited sample size from European ancestry exclusively, these genetic risk factors require further investigation.

A meta-analysis conducted in 2017 demonstrated a genetic link between PSP, frontotemporal degeneration (FTD) and CBD. Using CBD GWAS data from Kouri et al., FTD GWAS data from the International FTD-Genomics Consortium (IFGC) and publicly available PSP GWAS data from the NIA Genetics of Alzheimer’s Disease Storage Site (NIAGADS), this study was able to identify several SNPs in *MOBP*, replicating previous results [104]. Importantly, this study also identified a plethora of novel associations including SNPs at *CXCR4, EGFR* and *GLDC* genes [105]. Notably, these genetic associations were only between CBD and PSP, whereas the only overlap found between CBD and FTD was in SNPs tagging the *MAPT* haplotype [105].

A more recent study linked intermediate repeat expansions in *C9orf7* to CBD. Large hexanucleotide repeats in *C9orf72* have widely been proven to be the most common genetic risk factors for amyotrophic lateral sclerosis (ALS) and FTD. Genomic samples from 354 autopsy-confirmed CBD patients were screened for this repeat expansion and were found to have a 3.5-fold higher risk for CBD when compared to a control group [106]. While this study was able to recruit the largest number of CBD cases to date, future studies with a larger sample size are necessary to further validate these associations.

## 4. Future Directions

Rare neurological disorders have a significant impact in our healthcare system, with a progressively increasing burden of management. Understanding the genetic basis underlying disease etiology represents the first step towards dissecting disease mechanisms in affected individuals, predicting disease risk, onset and progression and establishing the foundation for succeeding functional studies. Generating genetic information for these diseases is key to having an accurate molecular diagnosis and clinical follow-up, as well as proper genetic counselling, and eventually paving the path to the design of new and more efficient therapies for these disorders. 

However, there are several aspects to be taken into consideration as we move forward: (1) Genetic research conducted in these diseases thus far has largely been centered on European ancestry populations. It is imperative that future efforts focus on unmasking the contribution of genetic variation in underserved and underrepresented populations to provide a broader picture of these conditions, and to identify possible genetic differences linked to disease presentation and course across ancestries. (2) Genetic research depends on large sample sizes, and powered studies can only be possible by promoting open science and connecting researchers worldwide in a collaborative framework. Democratization of data and resources is required. (3) A crucial step to gain biological insights underlying the clinical heterogeneity of these diseases is to generate deep clinical phenotypic data. Integration of these data as part of multimodal approaches is necessary to keep assembling this complex puzzle. (4) Genetics is only partially useful in isolation. The availability of additional data, including neuropathological and neuroimaging data, transcriptomics, epigenomics, metabolomics and proteomics affords the opportunity to further understand the genetic basis of variation in these measures, offering insights on disease subtyping and potential biomarkers.

## Figures and Tables

**Table 1:** Genetics of dystonia.

Dystonia Locus	Chr Region	Gene Identified	Phenotype	Inheritance	Protein Name	Protein Function
**Combined Dystonias (Dystonia-Plus Syndromes)**						
*DYT3*	Xq13.1	*TAF1*	X-linked dystonia-parkinsonism	XR	Multiple transcript system, TAF1	Core scaffold of transcription factor IID
*DYT5a*	14q22.1–q22.2	*GCH1*	Dopa-responsive dystonia	AD	GTP cyclohydrolase 1	Synthesis of tetrahydrobiopterin (BH4)
*DYT5b*	11p15.5	*TH*	Dopa-responsive dystonia	AR	Tyrosine hydroxylase (TH)	Synthesis of TH
*DYT11*	7q21–q31	*SGCE*	Myoclonus-dystonia	AD	Epsilon-sarcoglycan	Unknown
*DYT12*	19q13	*ATP1A3*	Rapid-onset dystonia-parkinsonism	AD	Na(+)/K(+)-ATPase alpha3 subunit	Sodium pump
*DYT15*	18p11	None	Myoclonus-dystonia	AD	N/A	N/A
*DYT16*	2q31.2	*PRKRA*	Early-onset dystonia-parkinsonism	AR	Protein kinase	Stress response
*-*	5p15.3	*SLC6A3*	Infantile parkinsonism-dystonia (dopamine transporter deficiency syndrome)	AR	Dopamine transporter (DAT)	Reuptake of dopamine from synapse
**Isolated or Primary Torsion Dystonias (not Covered in this Review)**						
*DYT1*	9q34	*TOR1A*	Generalized early-onset-limb dystonia	AD	TorsinA	Chaperone, ATP binding
*DYT6*	8p11.21	*THAP1*	Mixed-type dystonia	AD	THAP Domain Containing 1	Regulates endothelial cell proliferation
*DYT7*	18p	None	Adult-onset cervical dystonia	AD	N/A	N/A
*DYT13*	1p36.13–36.32	None	Craniocervical, laryngeal and limb dystonia	AD	N/A	N/A
*DYT17*	20p11.2–q13.12	None	Familial dystonia	AR	N/A	N/A
*DYT21*	2q14.3–q21.3	None	Adult-onset generalized or multifocal dystonia	AD	N/A	N/A
*DYT25*	18p11.21	*GNAL*	Adult-onset cranial-cervical dystonia	AD	Guanine nucleotide-binding protein G(olf) subunit alpha	Signal transduction within the olfactory neuroepithelium and the basal ganglia

XR: X-linked inheritance, AD: autosomal dominant, AR: autosomal recessive.

## Data Availability

Data sharing not applicable. No new data were created or analyzed in this study. Data sharing is not applicable to this article.
